# High-sensitivity optical to microwave comparison with dual-output Mach-Zehnder modulators

**DOI:** 10.1038/s41598-018-22621-1

**Published:** 2018-03-12

**Authors:** Mamoru Endo, Tyko D. Shoji, Thomas R. Schibli

**Affiliations:** 1 Department of Physics, The University of Colorado, Boulder, Colorado 80309–0390 USA; 2 JILA, NIST, and the University of Colorado, Boulder, Colorado 80309-0440 USA

## Abstract

We demonstrate the use of two dual-output Mach-Zehnder modulators (DO-MZMs) in a direct comparison between a femtosecond (fs) pulse train and a microwave signal. Through balanced detection, the amplitude-to-phase modulation (AM-PM) conversion effect is suppressed by more than 40 dB. A cross-spectrum technique enables us to achieve a high-sensitivity phase noise measurement (−186 dBc/Hz above 10-kHz offset), which corresponds to the thermal noise of a +9 dBm carrier. This method is applied to compare a 1-GHz fs monolithic laser to a 1-GHz microwave signal generated from photodetection of a free-running 500 MHz mode-locked laser. The measured phase noise is −160 dBc/Hz at 4-kHz, −167 dBc/Hz at 10-kHz, and −180 dBc/Hz at offset frequencies above 100-kHz. The measurement is limited by the free-running 500-MHz laser’s noise, the flicker noise of the modified uni-traveling carrier photodiode and the thermal noise floor, not by the method itself. This method also has the potential to achieve a similar noise floor even at higher carrier frequencies.

## Introduction

Great progress in the generation of ultra-low phase noise optical pulse trains and associated optical frequency-division (OFD)-based microwave generation^1–5^ has enabled not only novel approaches to radar applications^6^, but also very-long baseline interferometry^7^, high-speed analog-to-digital conversion^8^ and timing distribution at large-scale scientific facilities such as next-generation X-ray free-electron lasers^9–11^. A recent study showed the phase noise of an OFD-generated 12-GHz carrier reaching −173 dBc/Hz at a 10-kHz offset from the carrier^3^. Also, fiber-based optoelectronic microwave oscillators have the potential to produce a 10-GHz signal with −160 dBc/Hz at a 10 kHz offset, while only requiring several hundred meters of optical fiber without any reference^12^. Characterizing such low phase noise microwaves requires a complex apparatus and long acquisition times because typical mixer-based phase detectors and local oscillators lack sufficient sensitivity and a cross-spectrum method with two phase detectors is essential. While some phase noise analyzers have a cross-spectrum option, the acquisition times required for measuring the state-of-the-art ultra-low noise oscillators is of the order of days in order to remove the phase noise of the local oscillators or the electrical noise from the detection circuit. Therefore, in order for the cross-spectrum method to eliminate the residual detection noise, it is still important to have high sensitivity even with a single-channel phase detector. Due to the physics in oscillators, there is potential for optical femtosecond (fs)-pulse trains to generate microwave signals with much lower jitter than signals produced by electronic means^13^. This suggests that a direct comparison between an optical reference pulse train and a microwave signal could greatly improve the achievable sensitivity and the dynamic range of microwave phase noise measurements. Recently, optical delay lines^14–17^, Sagnac loop fiber interferometers^9,18^, and dual-output Mach-Zehnder modulator (DO-MZM)-based phase detectors^19^ have been demonstrated. When used as phase detectors, DO-MZMs are simple to calibrate, provide a direct link between optical and microwave oscillators and offer a high sensitivity. The DO-MZM phase detectors have been applied to timing synchronization between a microwave and a fs optical pulse^19^. In such applications, the bias voltage drift of the DO-MZM may spoil its long-term stability because this voltage directly introduces timing error between the signals, so a special bias control technique is required. However, in contrast to timing synchronization, the effect of the bias drift is not critical for phase noise characterization. In fact, the sensitivity error was less than 0.01 dB in our experiment for a typical measurement over a million averages (Supplement 1). Another concern regarding the DO-MZM phase detector is the shot-noise of the balanced detector that is used to extract the phase difference between the optical and the microwave signals, and the intrinsic flicker noise of the DO-MZM. Here, we overcome this limitation with cross-spectrum techniques (Methods).

Although fiber delay line methods have the ability to measure over a wide frequency range, they also require temperature-stabilized km-long optical fibers, which must be well-isolated from acoustic or mechanical vibrations. The use of telecom-band DO-MZMs can eliminate the difficulties associated with fiber delay lines. The nonlinear effects in the fiber can also be a concern for fs-sources because they typically limit the achievable dynamic range of fiber-based phase noise measurements. In contrast, DO-MZMs enable robust, high-sensitivity measurements. Furthermore, this method is largely insensitive to amplitude noise in both the microwave signal and the fs light source. In this regard, the proposed dual DO-MZM phase measurement setup is like an optoelectronic equivalent to a triple-balanced microwave mixer.

In this paper, we compare a fs pulse train from a free-running 1-GHz monolithic laser^20^ to a 1-GHz microwave signal generated by OFD of a free-running laser. DO-MZMs and balanced photodetectors suppress the amplitude-to-phase modulation (AM-PM) conversion effect by more than 40 dB in the phase detection, and cross-spectrum techniques suppress the associated shot-noise. Even without any external optical or electronic references, the noise floor of this method is −186 dBc/Hz above 10-kHz offset, which exceeds state-of-the-art cross-spectrum based fiber delay analyzer (OEWaves, microwave PNTS) by 10 dB^21^. With the free-running OFD, the measured phase noise of the 1-GHz microwave reaches −160 dBc/Hz at 4 kHz and −167 dBc/Hz at 10 kHz. These values are limited by the noise of the 500-MHz laser, the flicker noise of the modified uni-traveling carrier photodiode (MUTC)^22^ (−130 dBc/Hz at 1 Hz offset) and the thermal noise floor of the 50-Ohm system. Notably, the last two noise contributions do not depend on the carrier frequency. That is, even at higher carrier frequencies one could expect to reach very similar noise values and a comparable dynamic range, as none of the above was limited by the actual phase noise of the OFD.

## Result

### DO-MZM based phase detector

**Figure 1:**
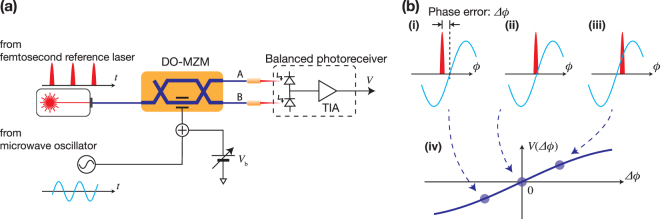
(**a**) Concept apparatus of a DO-MZM phase detector. (**b**) Phase error and discrimination signal. (i) (ii) and (iii) illustrate phase errors of < 0, = 0 and > 0, respectively. (iv) Shows the discrimination signal. Each blue circle corresponds to (i) to (iii). After a low-bandwidth phase lock, the residual voltage noise corresponds to the phase noise.

Figure 1(a) shows a conceptual diagram of the electro-optic phase detection method based on a single DO-MZM. As a timing reference, a low noise fs laser was used. The pulse train from this reference laser was fed into a DO-MZM, which was operated at the quadrature point with an appropriate bias voltage (*V*_b_). Although the bias voltage drift of the DO-MZM affects the phase sensitivity and AM-PM conversion coefficient (or AM sensitivity), in our experiment the drift effect was negligible (Supplement 1). For longer averaging times, one can use a second harmonic-pilot tone technique for active bias stabilization. The microwave signal under test drives the DO-MZM. Here, we assume that the microwave signal is identical to the repetition rate of the reference laser (or a harmonic of the repetition rate), but we allow for a phase difference ${\rm{\Delta }}\varphi$ (as shown in Fig. 1(b) for (i) ${\rm{\Delta }}\varphi  < 0$, (ii) ${\rm{\Delta }}\varphi =0$ and (iii) ${\rm{\Delta }}\varphi  > 0$). The output voltage of the balanced photoreceiver $V({\rm{\Delta }}\varphi )$ is proportional to ${\rm{\Delta }}\varphi$ and crosses zero as shown in (iv), which makes the phase detector insensitive to AM on the microwave signal and the reference femtosecond laser to first order. That is, when the discrimination signal is locked to the zero-crossing point (Fig. 1(b) (ii)), the output voltage of the balanced detector does not depend on the amplitude of the microwave or the reference laser. For example, when the peak-to-peak value of the signal from the balanced detector is 10 mV around 0 V with the full scale of the discriminator of 1 V, the AM suppression can be roughly estimated to be −40 dB. The sensitivity of this phase detector depends on the optical and microwave powers. Although with higher power one can obtain higher sensitivity, nonlinear effects and AM-PM conversion at the photodiodes or in the DO-MZM may ultimately limit the power scaling. Because the output voltage directly represents the phase fluctuation, the Fourier transform of it corresponds to the phase noise power spectral density (PSD). The calibration method is described in Supplement 1.

Although the DO-MZM phase detector has a high sensitivity, the achievable noise floor is limited by shot noise and the instrument noise, including the flicker noise of the DO-MZM, balanced photodetector, and amplifiers. To eliminate these sources of noise and to reach the thermal noise floor limit, a cross-spectrum method can be applied, which uses two statistically independent phase detectors (see Methods). Note that typical cross-spectrum measurements require two independent reference oscillators because the phase noise of the reference oscillators is typically worse than that of the device-under-test (DUT). When the phase noise of the reference is similar or better than that of the DUT, only one reference is required^23^. Here the phase noise PSD of the reference laser is much lower than that of the DUT, and therefore one reference laser is sufficient and allows for much faster data acquisition compared to traditional cross-spectrum measurements.

### Phase noise measurement

**Figure 2:**
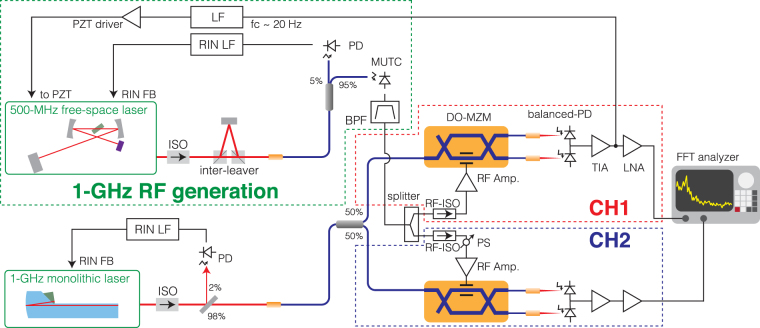
Schematic apparatus of the DO-MZM phase noise measurement with the cross-spectrum method. The dashed-green rectangle shows the 1-GHz RF signal generation via optical frequency division. The dashed-red and dashed-blue rectangles show the DO-MZM-based phase detectors for channel 1 and 2 (CH1, CH2). PD: photo detector; TIA: trans-impedance amplifier; LNA: low-noise amplifier; ISO: optical isolator; RF-ISO: RF isolator; MUTC: modified uni-traveling carrier photodiodes; BPF: 1-GHz band-pass filter; LF: loop filter.

For the DO-MZM method, an ultra-low noise monolithic fs laser with a fundamental repetition rate of 1 GHz^20^ was used as the timing reference. This relative intensity noise (RIN)-stabilized monolithic laser provided much lower phase noise than typical fs mode-locked lasers (the RIN spectra of the monolithic laser can be found in Supplement 1). For fiber and free-space lasers, the quantum noise limited performance is estimated to be at −170 dBc/Hz at 1 kHz and −190 dBc/Hz at 10 kHz offsets for 1-GHz carrier frequency^20^. As shown in Fig. 2, the output of the monolithic laser was coupled to a polarization-maintaining single mode fiber (PM-SMF), then split by a 50:50 PM-SMF coupler. Both branches were connected to DO-MZM phase detectors (CH1 and CH2) as described above. For the DUT, an optically generated 1-GHz microwave was used (see Methods). The 1-GHz microwave was split by a power splitter (Mini-circuits, MSC-2-11+), then each part was amplified by separate RF amplifiers (Mini-circuits, ZHL-2010+ and ZHL-1010+), and fed into the two DO-MZMs (JDSU, model 21014994). To suppress RF back reflections from the amplifiers, RF isolators (RF-ISOs) were installed after the RF splitter. A phase shifter (PS) was used for adjusting the phase difference between CH1 and CH2. Bias voltages for the DO-MZMs were adjusted to operate them at the quadrature points (not shown in Fig. 2). For optimizing the discrimination signal, the microwave power for the DO-MZMs were set to be approximately +20 dBm with RF attenuators (not shown in the figure. For optimization, see Supplement 1). The output from each DO-MZM was detected by balanced photodiodes (PD) (balanced PDs: each PD is Hamamatsu, G12180–003A). Both photodiodes generated a photocurrent of 1.9 mA. The balanced photocurrents were then amplified by transimpedance amplifiers (TIAs) and low-noise voltage amplifiers (LNAs). Then the PSDs were measured by a home-made two-channel FFT analyzer based on a commercial field-programmable gate array (FPGA) board with fast analog inputs (SETM lab, RedPitaya 14, see Supplement 1). To stabilize the microwave frequency to the reference monolithic laser, a loop filter (LF), piezoelectric actuator driver (PZT driver) and a PZT stack actuator were used to tune the 500-MHz laser cavity. Because the loop bandwidth was set to be below 20 Hz, the phase noise PSD above 200 Hz can be treated as free-running (out-of-loop) noise. For a cross-spectrum experiment, it is critically important that the two channels are in identical configurations but statistically independent. To achieve optimal isolation, separate power supplies were used for each channel. The phase noise PSD was also measured by a calibrated, commercial signal analyzer (SA: Keysight, PXA N9030A) to make sure our calibration was correct.

## Discussion

**Figure 3:**
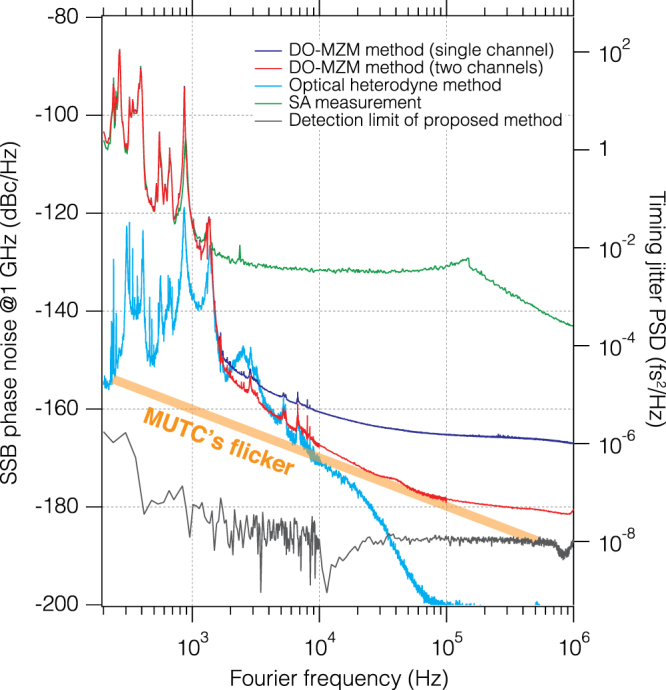
The results of phase noise measurement (left: SSB phase noise, right: corresponding timing jitter PSD). Green: measured by the commercial SA, Blue: single-channel DO-MZM method, Red: two-channel (cross-spectrum) DO-MZM method, Sky blue: optical heterodyne method, Black: Detection limit of the proposed method. The thick orange line is the flicker noise of the MUTC.

The SA data is shown as the green trace in Fig. 3. The phase noise PSD of a single channel DO-MZM channel is shown in blue (1,700,000 (1.7 M) averages). The red trace shows the cross-spectrum data (6.7 k averages below 10 kHz; 110 k averages between 10 kHz–100 kHz; 1.7 M averages above 100 kHz). It took approximately 1 hour to simultaneously acquire the blue and red traces. The DO-MZM and SA measurements agree well at frequency offsets below 1 kHz. Above 1 kHz, the green trace (by the SA) is limited by the instrument noise floor (~ −130 dBc/Hz). For a single DO-MZM (blue), the 1/*f* slope from 2 kHz to 10 kHz is caused by the flicker noise of the DO-MZM and the balanced photodiode (approximately −120 dBc/Hz at 1 Hz)^24^, and the noise floor above 10 kHz is due to the shot noise. These noise terms can be fully removed with the cross-spectrum method. The cross-spectrum trace (red) reaches −160 dBc/Hz at 4 kHz and −167 dBc/Hz at 10 kHz. The thermal noise floor (−180 dBc/Hz) is reached at 100 kHz. Note that carrier scattering in the MUTC with long pulse duration may also limit the noise floor of the OFD^25^. In this experiment, the pulse duration at the MUTC was less than 300 fs after a 1.5-m single mode fiber, and it corresponds to the scattering carrier contribution of −225 dBc/Hz, which is much smaller than that of the thermal noise of the 1-GHz microwave signal. The small feature around 40 kHz is caused by the RIN suppression servo bump of the 1-GHz monolithic laser. The thick orange line shows the flicker noise of the MUTC (−130 dBc/Hz at 1 Hz), which is typical for such detectors^3,13^. The measurement of this flicker noise is described in Supplement 1. The black trace shows the detection limit of cross-spectrum DO-MZM method (1.4 M averages below 10 kHz and 22 M averages above 10 kHz), measured by terminating the splitter’s input with a 50-Ohm terminator instead of the MUTC. This measurement represents the noise floor of a noiseless microwave oscillator with an ideal fs reference laser. The instrument noise floor at 10 kHz is approximately 55 dB better than the SA’s noise floor (−186 dBc/Hz). Although 22 M averages were used, corresponding to a 36-dB noise reduction, the noise floor only decreased by 20 dB. This is because the noise floor is limited by the thermal noise of the splitter, the RF-isolators and the terminator. Note that to obtain the black trace, the bias voltage was manually controlled to keep the DC value of the balanced detector to be zero because the acquisition time was more than 10 hours for this trace. However, usually this control is not required (Supplement 1). The dips in the black trace around 10 kHz and 800 kHz are due to cross-spectrum collapse^26^ and do not represent the real noise floor (see Methods).

We also measured the phase noise PSD in the optical domain (sky blue trace in Fig. 3) using the optical heterodyne method^27^. The peak around 2.5 kHz in the phase noise PSD corresponds to the servo bump from phase locking. This trace limits the phase noise PSD of the 1-GHz microwave. The residual 20 dB/decade ($1/{f}^{2}$) noise between 2 kHz to 10 kHz is due to the SESAM in the 500-MHz laser. The phase noise in the lower frequency region is due to acoustics and mechanical resonances of the 500-MHz free-space laser. This noise could be suppressed by stabilizing the laser to an optical reference, or by replacing the laser by a more stable source such as a second monolithic laser.

In conclusion, we demonstrated a novel, high-sensitivity photonic to microwave comparison method with two DO-MZMs and cross-spectrum analysis. With free-running OFD, the phase noise can be well explained by the optical domain measurement, flicker noise of the MUTC and the thermal noise floor. The noise floor of this method reaches −186 dBc/Hz above 10 kHz offset frequency. Further, we would like to point out that instrument noise floor is independent of the input frequency, and hence this technique would readily allow a sensitivity exceeding −180 dBc/Hz above a 10-kHz offset from a 10-GHz carrier. In this experiment, we only used one reference laser (a monolithic mode-locked laser) because the phase noise of the reference is sufficiently below that of the DUT. For state-of-the-art ultra-low phase noise oscillators (especially with low phase noise below 10-kHz offset), another monolithic laser as a second reference for CH2 could be employed, or the reference laser could be locked to a high-finesse reference cavity. By employing two reference lasers, the residual phase noise of the monolithic laser (especially for offset frequencies below 10 kHz, which is the most important range for ultra-low noise microwave oscillators) could be eliminated. That is, a true cross spectrum measurement is possible, though not required for most microwave sources. For very-long-term averaging (e.g. longer than 10 hours), the drifts of the bias voltages for the DO-MZMs could be a problem. To address this, a second harmonic pilot tone can be used to stabilize the bias voltage at the quadrature point^28^. This could also improve the sensitivity at lower offset frequencies.

An evident drawback of this method is that it can only be applied for specific frequencies (harmonics of the repetition frequency of the reference laser) within a tuning range of the reference laser (in our case, the tuning range of the monolithic laser is of the order of ±1 MHz for a 10-GHz carrier frequency). However, its ultra-high sensitivity compared to fiber-delay-based methods or commercial phase noise analyzers, such as Holtzworth HA7062C with option HX4920, enables the characterization of ultra-low noise, fixed frequency oscillators, which typically operate at well-defined frequencies. We believe that this method can be applied not only in the metrological field, but also for precision instrumentation equipment.

## Methods

### 1-GHz microwave generation from a 500-MHz free-space laser

The DUT microwaves were generated via photodetection of a home-built 500-MHz fs mode-locked laser as shown in Fig. 2 (“1-GHz microwave generation”). The details of this laser can be found in our previous paper^27^. The output was sent through a pulse inter-leaver with two polarizing beam splitters (PBSs) to suppress the 500 MHz fundamental carrier and enhance the second harmonic at 1 GHz. In our case, the 500 MHz fundamental signal was suppressed by more than 45 dB. The light was then coupled into a PM-SMF. 5% of the light was used for active suppression of the RIN using a photodetector and a RIN feedback loop filter (RIN LF). The pulse train was detected by an MUTC. MUTCs are suitable for photonic microwave generation because of their high saturation power and low AM-PM conversion coefficient^3,13,29^. The MUTC’s bias voltage was set to −12 V and the photocurrent was 5.0 mA when the incident optical power was 15 mW. These parameters were selected to minimize the AM-PM conversion effect of the MUTC for the 1-GHz carrier. The conversion ratio from RIN to phase noise was approximately −45 dB. A 1-GHz carrier power of +3.0 dBm was obtained, limited by the low microwave frequency. Higher RF powers could be extracted at higher microwave frequencies. In our experiment, the highest DUT frequency was limited by the DO-MZM’s bandwidth (~1 GHz), but this could be increased by replacing the commercial DO-MZMs used here with higher bandwidth modulators, which are readily available for the X to W bands. Since the sensitivity in this double DO-MZM setup is not limited by the modulators, comparable performance is expected at much higher carrier frequencies.

### Cross-spectrum method

With a single DO-MZM phase detector, the sensitivity is limited by shot noise, the electronic noise of the balanced photoreceiver, the flicker noise of the DO-MZM, the RF amplifiers and the FFT analyzer, which cannot be suppressed by simple averaging. The noise contributions from these sources can be eliminated in oscillator phase noise measurements through cross-spectrum measurement techniques^3,30^. The cross-spectrum is calculated by simultaneously measuring the phase noise of the device under test (DUT) with two independent setups and comparing the results. After averaging $m$ independent measurements, uncorrelated noise from each channel is reduced by a factor of $1/\sqrt{m}$ (e.g. 10,000 averages reduce uncorrelated noise by 20 dB). The correlated signal on the other hand, does not average away. Therefore, the effective signal-to-noise improves at the cost of longer measurement times.

Note that cross-spectrum methods can potentially underestimate phase noise PSD, especially for oscillators limited by thermal noise^26^. For instance, the anti-correlated thermal noise of the 100-ohm isolation resister in the Wilkinson splitter, which is used to divide the DUT microwave to two channels, can interfere with the actual phase (or thermal) noise of the oscillator. There are ongoing studies to develop methods to avoid this “cross-spectrum collapse” with a splitter in a cryostat^31^, or via novel splitter architectures^32^. In the data presented here, we were careful to identify or avoid such cross-spectrum collapse. The residual noise floor is limited by a cross talk of each channel or common mode noises.

## Electronic supplementary material

Supplementary Information
