# Supplementary material for: High-sensitivity optical to microwave comparison with dual-output Mach-Zehnder modulators
Source: Sci Rep. 2018 Mar 12;8:4388. doi: 10.1038/s41598-018-22621-1 (PMC5847530; doi:10.1038/s41598-018-22621-1)
Supplement: Supplementary file 1 — Supplementary Information [file 41598_2018_22621_MOESM1_ESM.docx]

Supplementary material for “High-sensitivity optical to microwave comparison with dual-output Mach-Zehnder modulators”

Mamoru Endo^1^, Tyko D. Shoji^1^, and Thomas R. Schibli^1,2,*^

1. Department of Physics, the University of Colorado, Boulder, Colorado 80309-0390, USA

2. JILA, NIST, and the University of Colorado, Boulder, Colorado 80309-0440, USA

*Corresponding author: trs@colorado.edu

In this supplementary material, we provide additional details on our experiment. The contents include the following: calibration method, DO-MZM bias voltage drift, self-measurement for MUTC’s flicker noise, laser RIN and an FPGA-based FFT analyzer.

Calibration method

The phase noise power spectrum density (PSD) $S_{\phi}\left( f \right)$ can be calculated by

|  | $S_{\phi}\left( f \right) [\mathrm{rad}^{2}/Hz]={KS}_{V}\left( f \right),$ | (S1) |
| --- | --- | --- |

where $K$ is the calibration coefficient and $S_{V}\left( f \right)$ is the PSD of the discrimination signal, which can be measured by an FFT analyzer. The calibration coefficient *K* can be obtained by measuring the slope of the discrimination signal, which corresponds to the effective carrier power of the signal. The discrimination signal $V(\Delta\phi)$ is a function of the optical power of the femtosecond pulse, the RF power to the DO-MZM, the half-wave voltage of the DO-MZM ($V_{\pi}$) and the gain of the low-noise amplifier ($g_{\text{LNA}}$), as given by

|  | ${V\left( \Delta\phi\right)[V]=g_{\text{LNA}}V}_{0}\sin\left( \frac{{\pi V}_{RF}}{{2V}_{\pi}}\sin\Delta\phi\right),$ | (S2) |
| --- | --- | --- |

where *V*_0_ is related to the optical power and the transimpedance gain of the photodetector, $V_{\text{RF}}$ is the peak-to-peak voltage of the microwave signal, and $\Delta\phi$ is the phase difference between the optical pulse and RF signal. When the discrimination signal is locked at a zero-cross point ($\Delta\phi\sim0$) with low locking bandwidth (e.g. less than 100 Hz), then for sufficiently small phase fluctuations ($\left| \delta\phi\right|\ll1 \text{rad}$), the slope of discrimination signal can be written as,

|  | $\frac{{\pi g_{\text{LNA}}V_{0}V}_{RF}}{{2V}_{\pi}} [\text{V/rad}].$ | (S3) |
| --- | --- | --- |

Then the phase noise PSD $S_{\phi}\left( f \right)$ is

|  | $S_{\phi}\left( f \right) \left[ \frac{\mathrm{rad}^{2}}{\mathrm{Hz}} \right]={{R\left( \frac{{2V}_{\pi}}{{\pi g_{\text{LNA}}V_{0}V}_{RF}} \right)}^{2}S}_{V}\left( f \right)$ | (S4) |
| --- | --- | --- |
|  | $=\frac{S_{V}\left( f \right)}{P_{\text{ eff}}},$ | (S5) |

where *R* is the load impedance (50 ohm) and $P_{\text{eff}}$ is the effective carrier power, defined as

|  | $P_{\text{eff}} \left[ W \right]=\frac{1}{R}\left( \frac{{\pi g_{\text{LNA}}V_{0}V}_{RF}}{{2V}_{\pi}} \right)^{2}.$ | (S6) |
| --- | --- | --- |

Because the discrimination signal can be represented in terms of its Fourier components, $P_{\text{eff}}$ can also be calculated by coherently summing the amplitudes of the harmonics as follows:

|  | $P_{\text{eff}}[dBm]=20\log\left( {10}^{0.05P_{1}[\text{dBm}]}+3\times{10}^{0.05P_{3}[\text{dBm}]}+\cdots\right),$ | (S7) |
| --- | --- | --- |

where $P_{1}$ and $P_{3}$ are the powers in the 1^st^ and 3^rd^ order harmonics of the discrimination signal in units of dBm, respectively. Here, we calculate $P_{\text{eff}}$ using eqn. (S7).

Fig. S1 (a) – (c) depict the discrimination signal at values of $V_{\text{RF}}$ equal to or greater than ${2V}_{\pi}/\pi$. When the signal is locked at a zero-crossing point (red circles), the residual noise corresponds to the phase noise. Fig. S1 (d) shows a graphical representation of the signal in the frequency domain. The red bars indicate the magnitudes of the Fourier components of the signal shown in Fig. S1 (a). To achieve a higher dynamic range, one can apply an RF amplitude greater than $V_{\pi}$ as shown in Fig. S1 (b) (${{\pi V}_{\text{RF}}}/{({2V}_{\pi})}=3$). In this regime, the signal becomes distorted and the power in the third harmonic $P_{3}$ is approximately equal to $P_{1}$ as shown in Fig. S1 (e). At the optimum microwave power, $P_{\text{eff}}$ is ~13 dB greater than $P_{1}$. In Fig. S1 (b), the red dashed trace shows the sinusoidal with $P_{\text{eff}}$, that is ${\pi{g_{\text{LNA}}V}_{0}V}_{RF}/({2V}_{\pi})\cdot sin \left( \Delta\phi\right)$. If the RF power is further increased (e.g. $\pi{V_{\text{RF}}}/{({2V}_{\pi})}=3.5$) as shown in Fig. S1 (c) and (f), the power in the third harmonic eventually saturates. While the extra RF power in Fig. S1 (c) could potentially improve $P_{\text{eff}}$ by a few dB, the enhanced RF input introduces additional zero-crossings in the discrimination signal (blue circles), with smaller effective discrimination slopes and a higher sensitivity to AM noise in the microwave signal. In this regime, one would have to be very careful to use the correct zero–crossing points. Due to the additional non-linear distortion and the absolute maximum voltage of the DO-MZM, we generally avoided that regime. The calculated effective carrier power $P_{\text{eff}}$ is shown in Fig. S1 (g), assuming $V_{\pi}=3.1 \text{V}$ for our DO-MZM. The $P_{\text{eff}}$ is normalized such that $P_{\text{eff}}=0 \left[ \text{dB} \right]$ at an input power of 5 dBm. The grey region in Fig. S1 (g) indicates the regime that was avoided. Note that the absolute value of $P_{\text{eff}}$ depends on the incident optical power on each photodiode, transimpedance amplifier (TIA) gain and low-noise voltage amplifier (LNA) gain. In our case, the microwave peak-to-peak voltage was ~6 V, or +20 dBm for a 50-ohm load impedance. An example of a measured discrimination signal is shown in Fig. S1 (h). This signal was recorded by slightly detuning the repetition rates of the 500-MHz laser. Fig. S1 (i) shows the frequency domain picture for a repetition rate detuning of about 3 kHz at *f*_rep_ = 500MHz, which leads to an effective $\Delta f$ of about 6 kHz for the 1-GHz carrier. The 1^st^ and 3^rd^ order harmonics had power levels of approximately –11 dBm and –12 dBm, respectively. Using eqn. (S1), the effective carrier power after the DO-MZM was calculated to be +0.3 dBm. Note that these traces were recorded at the point before the LNA with an added attenuator. The actual $P_{\text{eff}}$ is obtained by multiplying the trace by the LNA gain and the attenuation factor. The even-order harmonics appear in Fig. S1 (i) due to a slight misalignment in the bias voltage for the DO-MZM. Careful adjustment of the bias voltage can effectively eliminate the 2^nd^ harmonic peak and maximize the power in the 3^rd^ harmonic. Consequently, a process that minimizes the 2^nd^ harmonic peak could be used to automate the *V*_π_ adjustment.

Figure S1. The simulated and experimentally acquired discrimination signals. (a), (b) and (c): simulated discrimination signals for ${\pi V_{\text{RF}}}/{(2V_{\pi})}=$ 1, 3 and 3.5. In (b), the red dashed trace shows the effective carrier. (d), (e) and (f): Fourier components of the discrimination signal for (a), (b) and (c), respectively. (g) Calculated normalized effective carrier power $P_{\text{eff}}$as the function of input power to the DO-MZM (bottom) and ${{\pi V}_{\text{RF}}}/{({2V}_{\pi})}$ (top). The dashed lines correspond to ${{\pi V}_{\text{RF}}}/{({2V}_{\pi})}=1, 3 \text{and} 3.5$, respectively. (h): experimentally obtained discrimination signal. (i) Fourier components of (h) measured by a signal analyzer. Compared to the 1^st^ order carrier, the 3^rd^ harmonic clearly shows a 9-fold increased phase noise sideband power, which boosts the sensitivity of the DO-MZM.


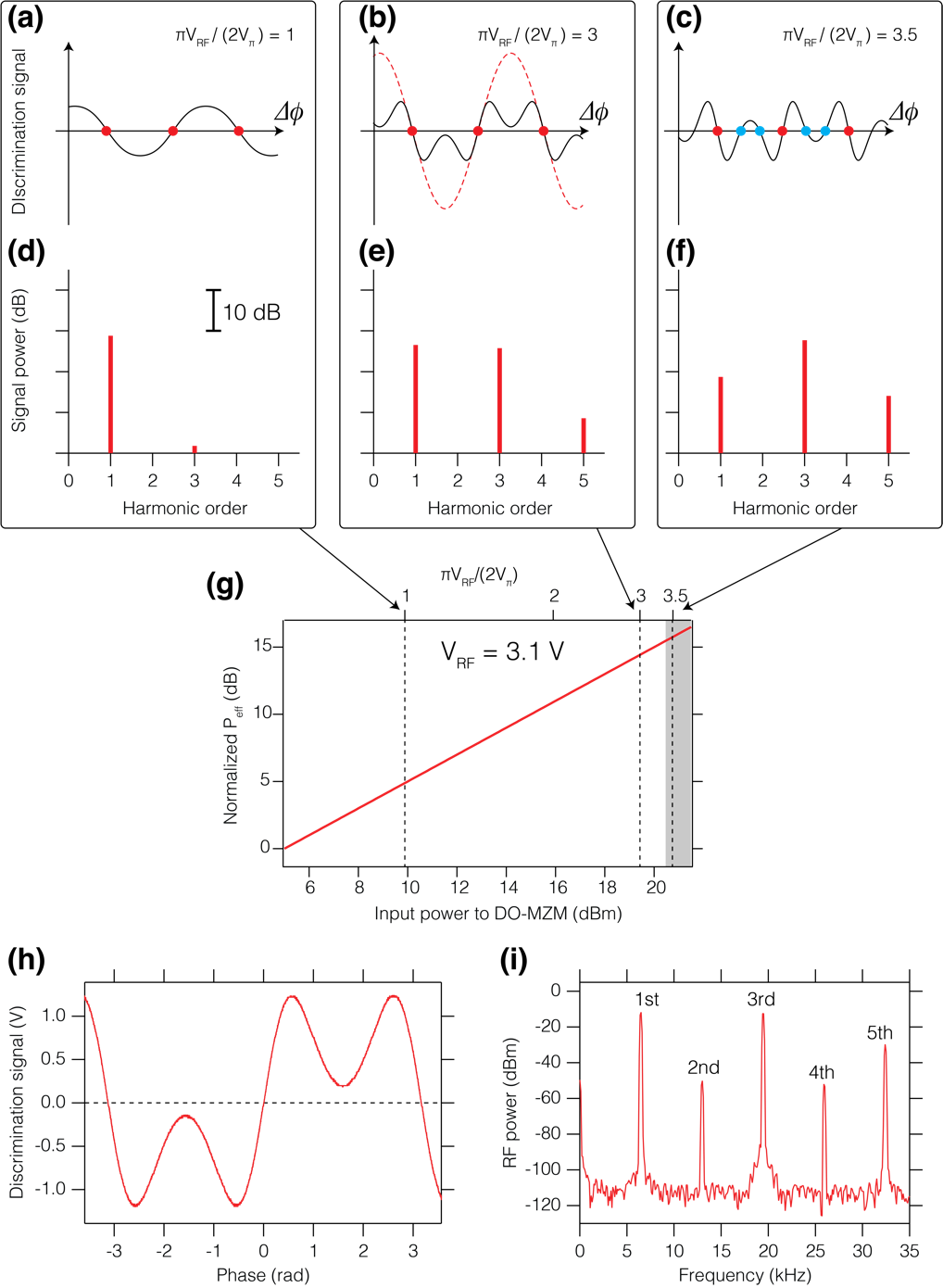


As a result, the single-side-band (SSB) phase noise PSD $\mathcal{L}\left( f \right)$is written as

|  | $\mathcal{L}\left( f \right)={\frac{1}{2}S}_{\phi}\left( f \right)=\frac{S_{V}(f)}{2P_{\text{eff}}}.$ | (S8) |
| --- | --- | --- |

In these equations, “$1/2$” is required because the $\mathcal{L(}f)$ is SSB phase noise PSD as defined in [S1].

Effect of DO-MZM bias voltage drift

One of the main concerns when using a DO-MZM as a phase detector is the bias voltage drift. Some applications, including timing synchronization between a fs-pulse and a microwave signal, require a bias voltage stabilization or cancellation method. However, in phase noise measurements, this effect can be negligible. We describe the effect of bias voltage drift below.

The bias voltage drift affects both the PM sensitivity and AM sensitivity of the microwave signal. In this section, we describe these effects as a function of the output voltage drift $\epsilon=\delta V/V$ of the discrimination signal as shown in Fig. S3(a).


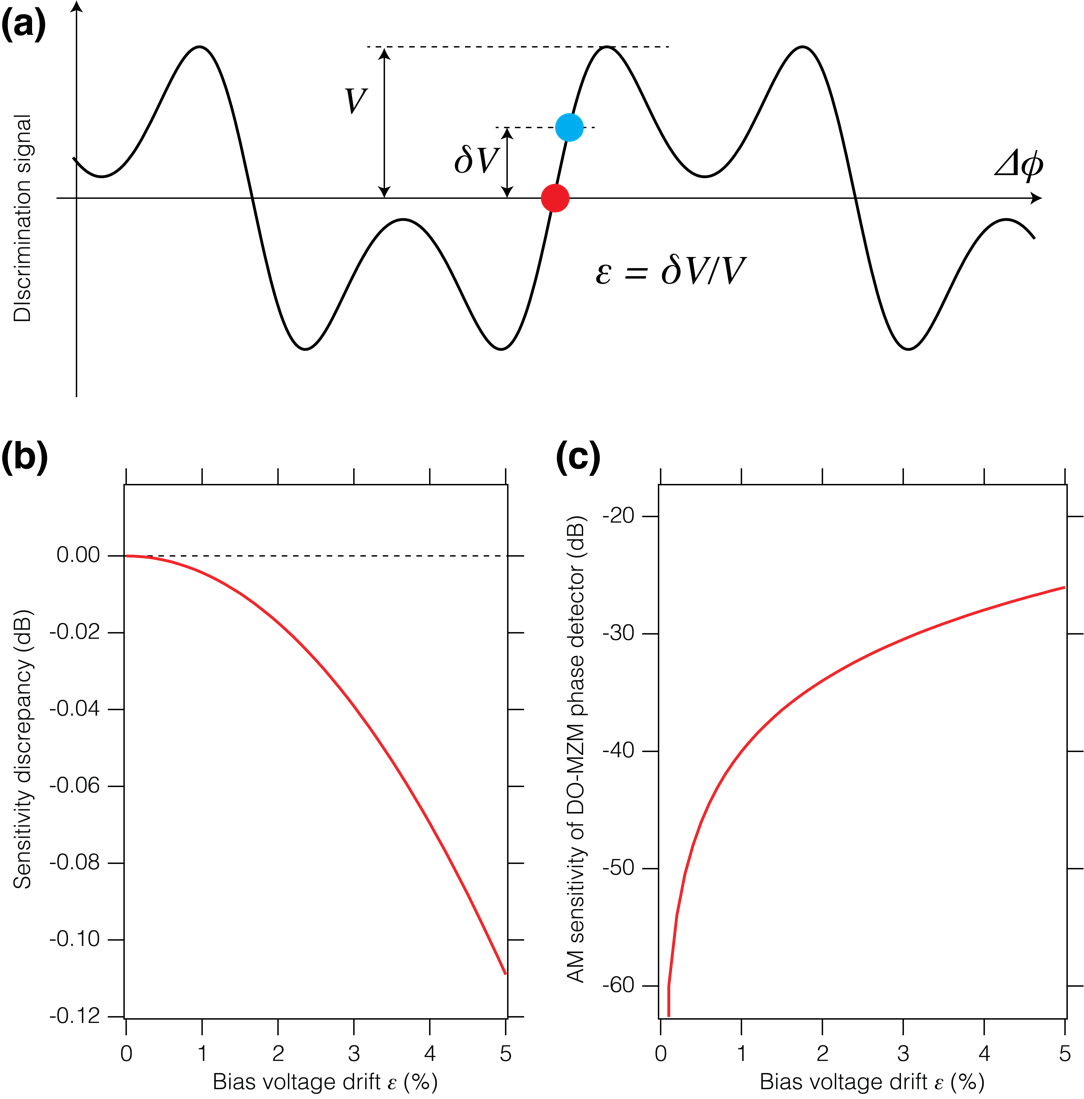


Figure S2. (a) effect of bias voltage drift of the DO-MZM phase detector. Red: ideal, blue: after a drift $\delta V$. (b) and (c): change in the PM and AM sensitivity of the DO-MZM phase detector as the function of bias voltage drift $\epsilon=\delta V/V$. In our experiment, $\epsilon\sim1\%$.

*1. PM sensitivity*

As mentioned above, the discrimination slope can be calculated as eqn. (S3). When the bias voltage changes by $\epsilon$ (from red circle to blue circle in Fig. S2(a)), the sensitivity can be calculated as followings:

|  | $\left. \frac{dV\left( \Delta\phi\right)}{d\Delta\phi} \right\vert_{\Delta\phi=\arcsin\epsilon}=g_{LNA}V_{0}\frac{{\pi V}_{RF}}{{2V}_{\pi}}\cos\left( \arcsin\epsilon\right)\cos\left( \frac{{\pi V}_{RF}}{{2V}_{\pi}}\sin\left( \arcsin\epsilon\right) \right)$ |  |
| --- | --- | --- |
|  | $=g_{LNA}V_{0}\frac{{\pi V}_{RF}}{{2V}_{\pi}}\sqrt{1-\epsilon^{2}}\cos\left( \frac{{\pi V}_{RF}}{{2V}_{\pi}}\epsilon\right).$ | (S9) |

Then the sensitivity error is

|  | ${\left. \frac{dV\left( \Delta\phi\right)}{d\Delta\phi} \right\vert_{\Delta\phi=\arcsin\epsilon}}/{\left. \frac{dV\left( \Delta\phi\right)}{d\Delta\phi} \right\vert_{\Delta\phi=0}}=\sqrt{1-\epsilon^{2}}\cos\left( \frac{{\pi V}_{RF}}{{2V}_{\pi}}\epsilon\right).$ | (S10) |
| --- | --- | --- |

The sensitivity error as a function of $\epsilon$ is shown in the Fig. S2(b), where ${{\pi V}_{\text{RF}}}/{({2V}_{\pi})}=3$. In our experiment, the maximum drift $\epsilon$ was less than 1% for the red trace (Fig. 3 in the main text), corresponding to a sensitivity error of only -0.004 dB, which is negligible.

*2. AM sensitivity*

The bias drift also causes an AM sensitivity change in the microwave signal. The first order AM sensitivity can be written as $20\log\epsilon$ and is shown in Fig. S2 (c). Because the AM noise of the microwave is sufficiently low (< -150 dBc/Hz after RIN stabilization of the 500-MHz laser), the noise contribution from this effect is approximately -190 dBc/Hz, which can also be neglected.

Fundamental limits of microwave generation


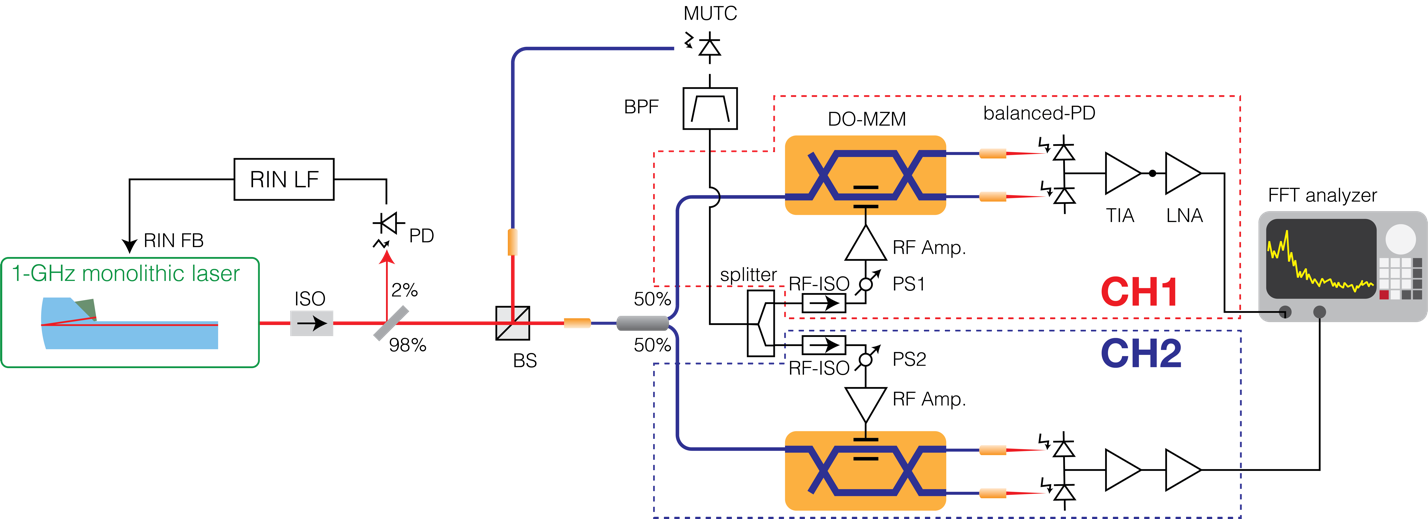


Figure S3. The schematic apparatus of self-measurement. PS1 and PS2: phase shifters to adjust to zero-crossing points for each channel.

Fig. 3 in the main text shows the flicker noise of the MUTC (indicated by the thick orange line). To reliably identify and characterize this flicker noise, a modified DO-MZM measurement setup was conceived. Figure S3 shows the schematic apparatus for this measurement. By using the same monolithic laser for both the optical reference and the 1-GHz microwave generation, the laser’s phase noise is common mode and therefore cancels out in the measurement. The remaining noise comes from the measurement noise floor imposed by technical limitations of the setup, such as noise from the optical fibers and excess noise from the MUTC, including flicker, thermal noise and AM-PM conversion. Other technical noise includes finite RIN rejection, AM-PM conversion in the balanced PIN diodes and noise from the subsequent TIAs and LNAs that are used to detect the light after the DO-MZMs.


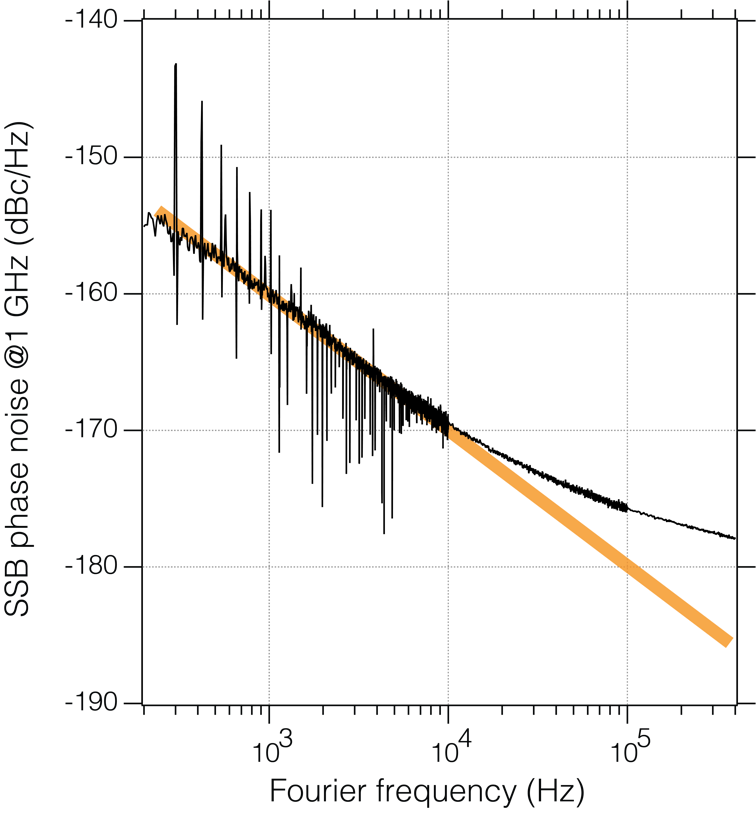


Figure S4. Self-measurement result. Black: measured cross-spectrum. Orange: line for $1/f$ (-130 dBc/Hz at 1 Hz)

In this modified setup, the light from the monolithic laser was divided into two branches via a beam splitter (BS). One branch was used as a reference for the DO-MZM phase noise detector and the other branch was used for microwave generation. The optical power on each diode of the balanced detectors was 0.5 mW. The microwave power at 1 GHz was set to be 0 dBm. The results are shown in Fig. S4 (22k averages at offset frequencies bellow 10 kHz; 360k averages at 10 kHz – 100 kHz; and 5.7M averages above 100 kHz). The $1/f$ slope at frequencies below 10 kHz is due to the MUTC flicker noise (-130 dBc/Hz at 1 Hz). The measured flicker noise is at a reasonable level for this type of MUTC [S2]. At frequencies higher than 100 kHz, the trace is limited by the thermal noise floor (-177 dBc/Hz for 0 dBm carrier power).

Laser RIN

The RIN spectra of the 1-GHz monolithic laser are shown in Fig. S5. The 500-MHz free-space laser has almost same value and structure.


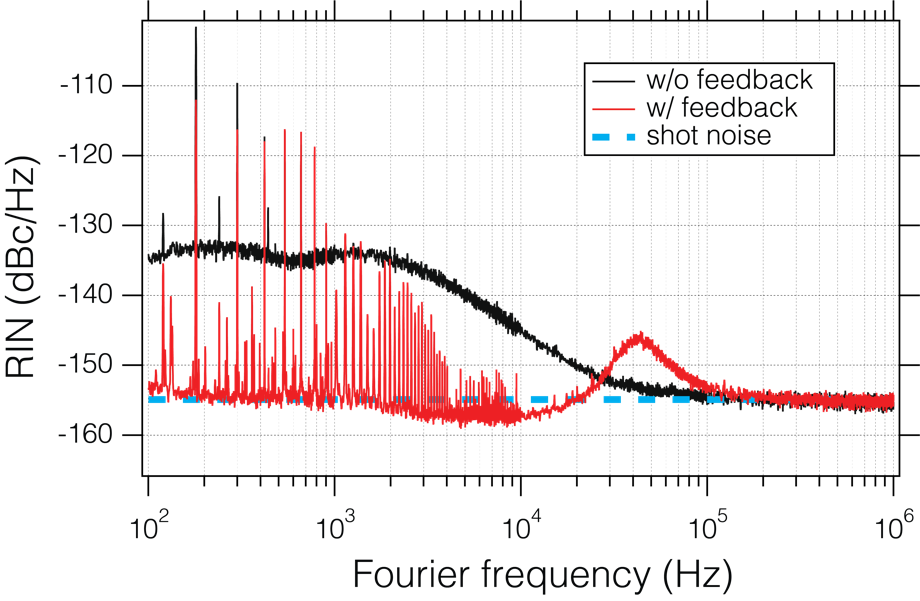


Figure S5. RIN spectra of the 1-GHz monolithic laser. Black: without RIN stabilization, Red: with RIN stabilization, Dashed sky blue: shot noise level.

Cross-spectrum FFT analyzer

The major drawback of cross-spectrum measurements is the long averaging time. Several research groups have developed field-programmable gate array (FPGA)-based cross-spectrum analyzers to overcome the limitations of commercial cross-spectrum analyzers, mainly relating to averaging speeds and simultaneous full frequency coverage [S3,S4]. We modified a commercially available FPGA board with fast analog inputs (RedPitaya, V1.2, now called STEM Lab, RedPitaya 14) to acquire and calculate the cross-spectrum for faster overall acquisition times. The original analog frontend of the FPGA board employed a dual operational amplifier (OPAMP) in a single chip to transform the input impedance for both channels. In cross-spectrum measurements, the cross-talk between the two channels limits the achievable noise suppression. For example, cross-talk of -50 dB limits the maximum achievable noise reduction to 25 dB, which corresponds to 100k averaged traces. We removed this OPAMP and added a new frontend with much better isolation and added anti-aliasing filters with 1.9-MHz corners (Mini-circuits, BLP-1.9+). Thanks to this modification, the cross-talk was improved from -50 dB to -80 dB. Thus, the maximum noise reduction of the cross-spectrum method became ~40 dB, which allowed for averaging over 100M traces.


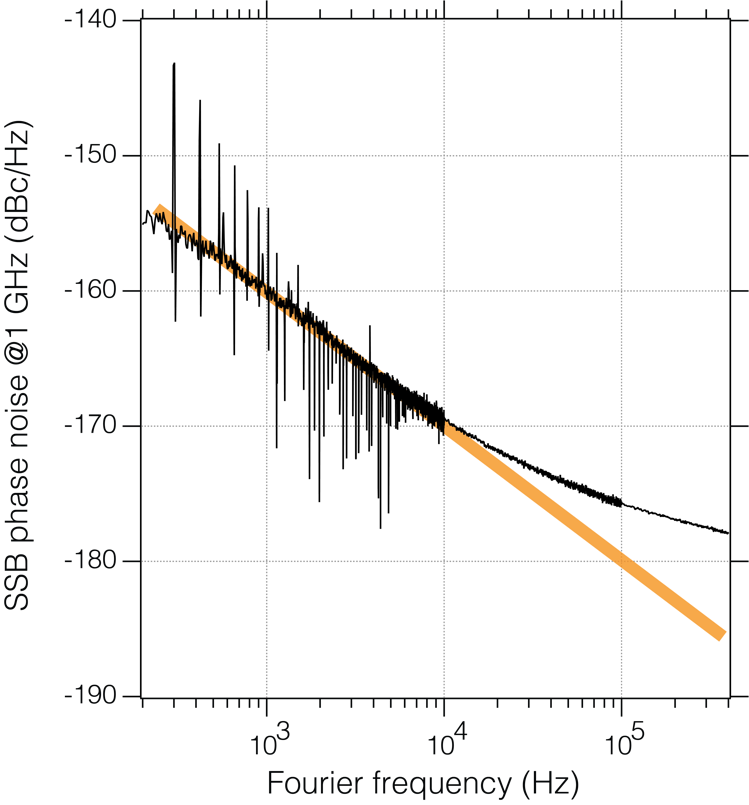


Figure S3. The result of self-measurement. Black: experimental data. Spikes are caused by electrical noises from the RF amplifier and the power supplies. Orange line: MUTC’s flicker noise (-130 dBc/Hz@1 Hz)

The averaging speed of this configuration is entirely limited by the desired resolution bandwidth (e.g. 4000 averages/s for a RBW of 4 kHz etc.). The data acquired by this FPGA-based cross-correlator agrees well with data acquired by a commercial two-channel FFT analyzer (HP, 89410A).

**References**

1. *IEEE Std 1139-2008*,. *IEEE* (IEEE, 2009). doi:10.1109/IEEESTD.2008.4797525
2. Fortier, T. M. *et al.* Photonic microwave generation with high-power photodiodes. *Optics Letters* 38, 1712–1713 (2013).
3. Fleischmann, P., Mathis, H., Kucera, J. & Dahinden, S. Implementation of a Cross-Spectrum FFT Analyzer for a Phase-Noise Test System in a Low-Cost FPGA. *International Journal of Microwave Science and Technology* 2015, 1–7 (2015).
4. Xie, X. *et al.* Photonic microwave signals with zeptosecond-level absolute timing noise. *Nature Photon* 11, 44–47 (2017).
